# The Effects of Dietary Pattern during Intensified Training on Stool Microbiota of Elite Race Walkers

**DOI:** 10.3390/nu11020261

**Published:** 2019-01-24

**Authors:** Nida Murtaza, Louise M. Burke, Nicole Vlahovich, Bronwen Charlesson, Hayley O’ Neill, Megan L. Ross, Katrina L. Campbell, Lutz Krause, Mark Morrison

**Affiliations:** 1 Faculty of Medicine, Translational Research Institute, University of Queensland Diamantina Institute, Brisbane, QLD 4102, Australia; nida.murtaza@uqconnect.edu.au (N.M.); l.krause@uq.edu.au (L.K.); 2 Centre for Exercise and Nutrition, Mary MacKillop Institute for Health Research, Australian Catholic University, Melbourne, VIC 3000, Australia; meg.ross@ausport.gov.au; 3 Australian Institute of Sport, Canberra, ACT 2617, Australia; Nicole.Vlahovich@ausport.gov.au (N.V.); bronwen.charlesson@outlook.com (B.C.); 4 Faculty of Health Sciences and Medicine, Bond University, Robina, QLD 4226, Australia; haoneill@bond.edu.au (H.O.N.); kcampbel@bond.edu.au (K.L.C.)

**Keywords:** stool microbiome, diet, race walkers, training

## Abstract

We investigated extreme changes in diet patterns on the gut microbiota of elite race walkers undertaking intensified training and its possible links with athlete performance. Numerous studies with sedentary subjects have shown that diet and/or exercise can exert strong selective pressures on the gut microbiota. Similar studies with elite athletes are relatively scant, despite the recognition that diet is an important contributor to sports performance. In this study, stool samples were collected from the cohort at the beginning (baseline; BL) and end (post-treatment; PT) of a three-week intensified training program during which athletes were assigned to a High Carbohydrate (HCHO), Periodised Carbohydrate (PCHO) or ketogenic Low Carbohydrate High Fat (LCHF) diet (post treatment). Microbial community profiles were determined by 16S rRNA gene amplicon sequencing. The microbiota profiles at BL could be separated into distinct “enterotypes,” with either a *Prevotella* or *Bacteroides* dominated enterotype. While enterotypes were relatively stable and remained evident post treatment, the LCHF diet resulted in a greater relative abundance of *Bacteroides* and *Dorea* and a reduction of *Faecalibacterium.* Significant negative correlations were observed between *Bacteroides* and fat oxidation and between *Dorea* and economy test following LCHF intervention.

What are the new findings?

➢ The gut microbiota profiles of endurance race walkers could be separated into two principal “enterotypes.”
➢ The LCHF diet is associated with a significant reduction in the relative abundance of *Faecalibacterium* spp. and an increase in the relative abundance of *Bacteroides* and *Dorea* spp.
➢ The relative abundance of some bacterial taxa was correlated with measures of athlete performance and metabolic capacity.

How might it impact on clinical practice in the foreseeable future?

Dietary patterns appear to exert a subtle but meaningful impact on the gut microbiota of elite endurance race walkers. In particular, there appears to be a plausible link between a LCHF diet, the gut microbiome and impairments in exercise capacity, which may be monitored and managed to improve exercise economy and performance

## 1. Introduction

The gut microbiome is now widely recognised to be a functional and dynamic interface between host genetics, environmental and lifestyle choices. A virtual plethora of observational and case-control studies have investigated and reported on the variations in the gut microbiome of healthy subjects compared to those afflicted with acute, chronic and non-transmissible diseases [1,2,3,4]. But relatively few studies to date have investigated the structure-function relationships of the gut microbiome of elite athletes, despite their remarkable physiology and metabolism compared to mainstream (regular healthy) members of the community. Clarke et al. [5] compared the gut microbiome of professional Irish rugby players with mainstream subjects of either matched BMI (>25) or average BMI (<25). Rugby players possessed greater bacterial diversity compared with both non-athlete groups, which could be readily linked to the greater intake of dietary protein measured for these athletes. More recently, metagenomic and metabolomics analyses of these same cohorts have shown that the gut microbiota of rugby players were enriched for pathways involved in amino acid and carbohydrate metabolism and possessed greater amounts of faecal short-chain fatty acids (SCFA) as compared to the sedentary controls [6]. Peterson et al. [7] have also compared the gut microbiota of professional cyclists and category 1 level (amateur) cyclists. These studies revealed the presence of three taxonomic clusters or “enterotypes” for all the cyclists: either *Prevotella* or *Bacteroides* dominated or a mixed taxa cluster. An increased abundance of *Methanobrevibacter smithii* was also evident in professional cyclists. Collectively, these studies confirm that the gut microbiota profiles of elite athletes are different to those of mainstream and/or non-elite control subjects and that diet might be a principal driver of these differences.

Typically, elite endurance athletes follow special dietary practices, particularly during periods of specialised training or competition preparation, to benefit from exercise-nutrient interactions that underpin adaptation and performance [8]. In the parent ‘Supernova study 1′, Burke et al. [9] investigated aspects of endurance capacity in a cohort of elite race walkers who followed one of three popular dietary approaches to a 3-week block of intensified training: a ketogenic Low Carbohydrate High Fat diet (LCHF) or diets with continuous or periodised exposure to high carbohydrate availability (HCHO or PCHO, respectively). Athletes consuming HCHO and PCHO diets were found to have improved exercise economy (increased speed achieved for a given oxygen utilisation) and race performance (event lasting ~40 min) compared to those athletes consuming a LCHF diet. This suggests that the latter dietary pattern, which achieved significant alterations in host physiology (i.e., a major increase in capacity for fat oxidation) is not conducive for performance of endurance exercise conducted at sustained higher intensities, where oxygen delivery to the muscle becomes limiting.

Given the scarcity of information on baseline (BL) gut microbiome profiles of elite athletes and the effect of dietary changes on the gut microbiota in such cohorts, the aim of the current investigation was to characterise the stool microbiome profiles elite race walkers that participated in the Supernova 1 study, using samples collected before and after undertaking 3 weeks of intensified training while following different dietary programs under rigorous study control. We hypothesised that the BL profiles might share features of the microbiota previously described in endurance athletes (cyclists) but that this might change in response to the radical dietary changes implicit in a ketogenic LCHF diet.

## 2. Materials and Methods

### 2.1. Study Design and Sample Collection

A detailed description of the experimental design and physiological measures performed as part of the Supernova 1 study was provided by Burke et al. [9] along with justification for each of the dietary interventions [8]. Briefly, a total of 21 male participants (aged 20–35 years), all of whom met International Association of Athletics Federations (IAAF) standards for international race experience, were accepted into the study and the anthropometric details of the subjects enrolled in this study are provided in Supplementary Table S1. The dietary interventions were conducted over two separate training camps in November 2015 (*n* = 10) and January 2016 (*n* = 19) with 8 athletes recruited into both camps. During their 3-week training camp, the athletes were assigned to the specific diets according to their beliefs in the potential effect on their performance to either a diet high in carbohydrate availability (HCHO; *n* = 9) comprised of 60% of caloric intake from carbohydrate, CHO (~8.5 g/kg body mass (BM)/day), 16% protein (~2.1 g/kg BM/day), 20% fat; a diet with periodised carbohydrate availability (PCHO; *n* = 10) of the same macronutrient composition as HCHO but periodised in consumption across the day and throughout the week, so to support different training sessions with a high and low CHO availability; and a ketogenic low carbohydrate-high fat diet (LCHF; *n* = 10) that was comprised of 78% fat, 17% of protein (~2.2 g/kg BM/day) and <50 g/day of carbohydrate content (~3.5% energy). Stool samples were collected from the athletes at the beginning and end of the 3-week training-diet intervention period using the OMNIgene stool collection and preservative kit. Physiological measures such as VO_2peak_ and walking economy, 10 km race time, 25 km long walk time, respiratory exchange ratio and fuel oxidation rates were measured in the Supernova 1 study by Burke et al. [9]. These data are used for the correlation analysis.

### 2.2. Stool Microbiota Analysis

Stool microbiome profiles were assessed using 16S rRNA gene amplicon sequencing using the Illumina MiSeq platform at the University of Queensland’s Australian Centre for Ecogenomics. Genomic DNA was extracted from all the stool samples using the repeated bead-beating procedure for cell lysis and an automated column-based DNA purification procedure (Maxwell^®^ 16MDx system, Promega Corporation, WI, USA). The PCR amplicon libraries (V6-V8) were produced using the Bacteria/Archaea specific primers 926-forward and 1392-reverse [10], which had been modified to include overhang adapters compatible with Nextera Index PCR XT kit (Illumina Corp., San Diego, CA, USA) to produce bar-coded amplicons for individual samples. The bar-coded amplicons were purified, quantified and subsequently pooled in equimolar quantities as described by Shanahan et al. [10].

The Quantitative Insights Into Microbial Ecology (QIIME) software package on an Ubuntu Linux virtual machine (v5.0.12) (Canonical, London, UK) was used to demultiplex and perform quality control checking, paired-end-merging and filtering of the sequence data, with a minimum quality score of 20 set as the acceptability threshold [11]. The sequences were then clustered into Operational Taxonomic Units (OTUs) using a threshold setting of 97% sequence identity and the open reference OTU picking method was used to assign sequences to their respective OTUs according to the Greengenes database version 75. Any candidate OTUs that were not identified as Bacteria or Archaea, and/or OTUs that comprised <0.01% of the total sample sequence count were removed from further analysis. USEARCH 6.1 was also used for reference-based chimera detection and any candidate chimeric sequences were removed [12]. One sample was removed during QIIME analysis due to the low number of reads. A rarefied subsampled OTU table was generated by random sampling to the minimum read count (*n* = 6861) and these data were used to generate taxonomy plots from phylum to genus levels and used to calculate alpha- and beta-diversity metrics. Alpha diversity was measured by Shannon’s and Simpson’s indices. Beta diversity was examined by weighted and unweighted UniFrac distances and used to ordinate samples by Principal Coordinate Analysis (PCoA). Multivariate statistical methods Redundancy Analysis (RDA) and Anosim were also used to identify associations between microbial community composition and diet. Individual taxa discriminatory for the different dietary patterns were identified by: Linear Discriminant Analysis (LDA) Effect Size (LEfSe) analysis performed within Calypso version 6.4 (San Francisco, CA, USA) [13] and sparse Partial Least Squares Discriminant Analysis (sPLS-DA) analysis via the Mixomics mixMC: multivariate data analysis framework [14] also available via Calypso version 6.4. Mixed effect linear regression (MELR) analysis via the repeated measures framework in Calypso was used to identify taxa significantly different between the groups. GraphPad Prism (version 7, GraphPad Software, San Diego, CA, USA) was used to perform the paired *t*-test to further confirm the statistical significance of the relative abundance data for taxa identified from MELR analysis. GraphPad Prism (version 7) was also used to perform the Spearman’s correlation between the individual taxa and performance measures. The significance level was set to *p* < 0.05 for all the analysis. Correction for multiple testing by false discovery rate (FDR) with values < 0.05 were considered significant in MELR analysis.

### 2.3. Ethics Approval, Clinical Trial Registration and Availability of Data

All participants were informed and consented to do this research and the study was approved by the Ethics Committee of the Australian Institute of Sport (AIS, no. 20150802) and has been registered as a clinical trial, assigned the number ACTRN12618001529235 by the Australian New Zealand Clinical Trials Registry (ANZCTR). The study is registered in European Nucleotide Archive (ENA) with the primary accession number PRJEB29450 and the DNA sequence data generation and analyses performed under UQ-HREC 2015001965.

## 3. Results

Burke et al. [9] reported that race walkers who consumed the LCHF diet during intensified training achieved a substantial increase (~tripling) in their rates of whole-body fat oxidation during exercise compared with BL, with peak rates of ~1.6 g/min (and up to 2 g/min in some individuals) being the highest reported values in the literature. However, this was associated with a reduction in exercise economy (increased oxygen cost to achieve the same walking speed) and a failure to improve 10 km race performance (change = 1.6% slower (90% CI = −8 to +5%) despite achieving an equal increase of 3–7% in aerobic capacity over the training block as the other groups. Conversely, athletes consuming either the HCHO (*n* = 8) or PCHO (*n* = 10) diets showed improvements in exercise economy and athlete performance as reported by Burke et al. (mean race improvements = 6.6% (4–9%) and 5.3% (3–7%), respectively). These differences in athlete physiology and performance in response to diet provide a unique opportunity to examine whether and how the gut microbiome of these athletes is affected by diet and/or can be associated with changes in athlete physiology and performance.

### 3.1. Bacteroides- or Prevotella-Enterotypes Are Predominant in Elite Race Walkers

**Figure 1:**
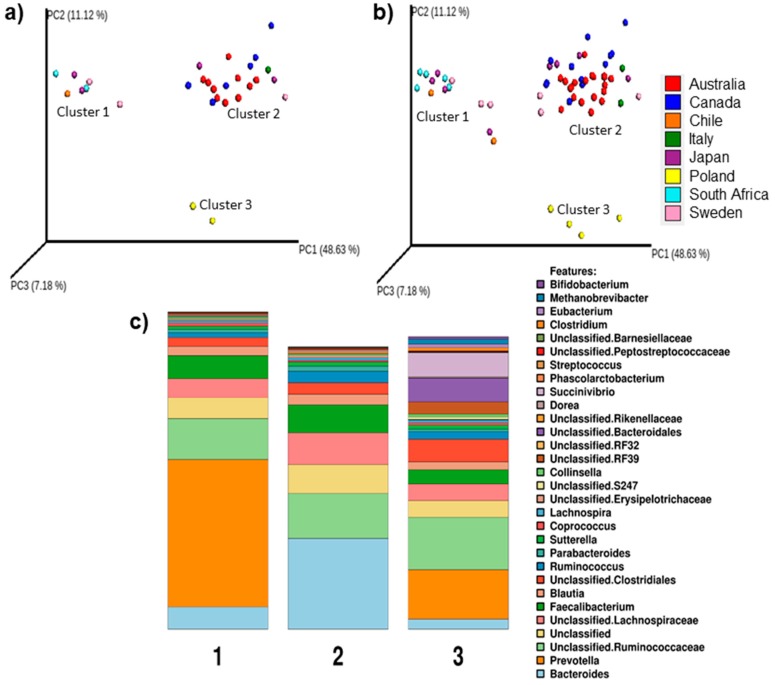
Principal coordinates analysis (PCoA) of the weighted Unifrac distances produced from the stool samples of athletes collected at baseline only (**a**); and combined with their matching stool samples collected after the 3-week diet-training intervention period (**b**). The individual samples are coloured coded according to the athlete’s country of origin. The athletes are separated into three distinct clusters and importantly, this clustering did not appear to be disrupted in response to the diet consumed during the training period. The first three Principal Coordinates and the amount of variation each explains are shown (PC1, PC2, PC3). (**c**) The profiles of the predominant taxa present in the baseline stool samples of athletes with either a *Prevotella-*dominant (P, cluster 1; *n* = 7), a *Bacteroides-*dominant (B, cluster 2; *n* = 20) or a Firmicutes-dominant (F, cluster 3; *n* = 2) “enterotype”.

Sample ordination by PCoA revealed that 28/29 athletes at BL could be separated into two distinct clusters, while 1 athlete showed a clear separation from both (Figure 1a). The genus-level taxonomic profiles for the three clusters at BL are shown in Figure 1c, with 7/29 athletes found to possess a “*Prevotella*-predominant” cluster and 20/29 athletes being “*Bacteroides*-dominant.” The remaining two points on the PCoA plot were from the same athlete who participated in both training periods and was a “Firmicutes-dominant” cluster with a remarkable level of methane-producing and succinate-utilizing microbes (*Methanobrevibacter* and *Succinivibrio*). Furthermore, these clusters were sustained throughout the intensified training and diet-intervention period, as shown when these profiles were included in the beta diversity analysis (Figure 1b). As noted in the Methods, a number of athletes participated in both camps, so the stool microbiota profiles of the two BL samples for these athletes were compared to each other. A combination of RDA and Anosim analyses as well as the Shannon diversity measures for the matched samples showed no remarkable or significant differences in these profiles. As such, the length of time between camps and the return of these athletes to their habitual diet was considered to be sufficient to ensure there were no carryover effects from the prior camp (and dietary intervention). In summary, the stool microbiomes of virtually all these elite athletes could be differentiated into either a *Prevotella* or *Bacteroides-*dominant enterotype and the dietary interventions and intensive training period had limited influence on the stability of these enterotypes.

### 3.2. HCHO and PCHO Diets Result in Subtle but Distinct Alterations in Firmicutes Lineages

HCHO (*n* = 8 athletes) and PCHO (*n* = 10 athletes) diets resulted in only minor, subtle changes in microbial community composition. RDA and Anosim did not identify any significant associations between microbial community composition and HCHO and PCHO diets and no differences in alpha diversity were observed between BL and post dietary interventions. Furthermore, the HCHO and PCHO diets did not result in any significant changes in the relative abundance of any specific taxa once adjustments were made for multiple testing using false discovery rate according to MELR repeated measures analysis.

The less stringent tests LefSe and sPLS-DA did identify a restricted range of bacterial taxa affected by these diets. LefSe found an increase of *Clostridiales,* in particular, *Ruminococcaceae*, *Coprococcus* spp. and *Akkermansia muciniphila* in athletes consuming the PCHO diet relative to their BL profiles and an increase of *Clostridiaceae*, *Lachnospiraceae* and *Ruminococcaceae* in athletes consuming the HCHO diet (Supplementary Figure S1a,b).

**Figure 2:**
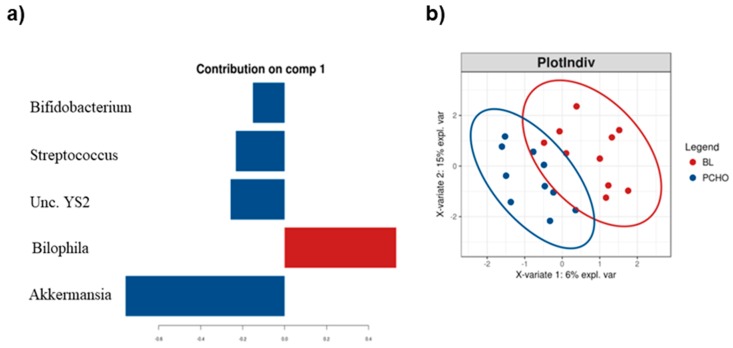
(**a**) Genera differentiating between the stool microbiota profiles of athletes at baseline (BL, red) and after their consumption of the Periodised Carbohydrate diet (PCHO, blue) identified by sPLS–DA; (**b**) The sPLS-DA ordination plot of these same data for each athlete, the ellipsoids represent 95% confidence intervals for each sampling period.

The sPLS-DA suggested that OTU’s assigned to *Unclassified (Unc.) YS2*, *Akkermansia, Bifidobacterium* and *Streptococcus* spp. were increased whereas *Bilophila* was decreased following consumption of the PCHO diet (Figure 2).

**Figure 3:**
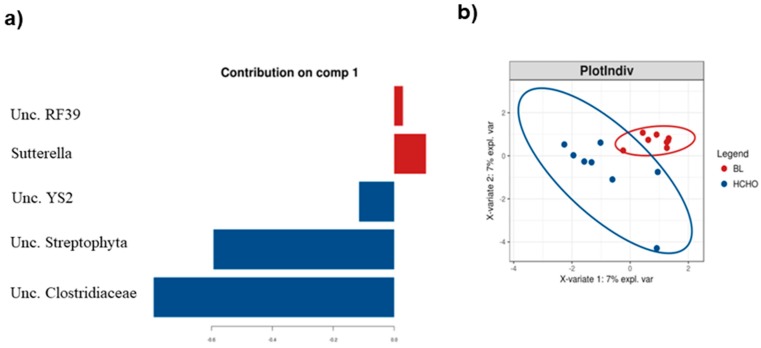
(**a**) Genera differentiating between the stool microbiota profiles of athletes at baseline (BL, red) and after their consumption of the High Carbohydrate diet (HCHO, blue) identified by sPLS–DA; (**b**) The sPLS-DA ordination plot of these same data for each athlete, the ellipsoids represent 95% confidence intervals for each sampling period.

The relative abundances of *Unc. YS2, Streptophyta* and *Clostridiaceae* were all increased in athletes who consumed the HCHO diet compared to their BL samples, whereas OTUs assigned to *Unclassified RF39* and *Sutterella* spp. were enriched and discriminatory of their BL samples (Figure 3). Collectively, these results show that the effects of consuming a carbohydrate-rich diet in these athletes was subtle and principally restricted to members of Firmicutes lineage.

### 3.3. The Low Carbohydrate High Fat Diet Results in More Profound Effects on the Gut Microbiota

**Figure 4:**
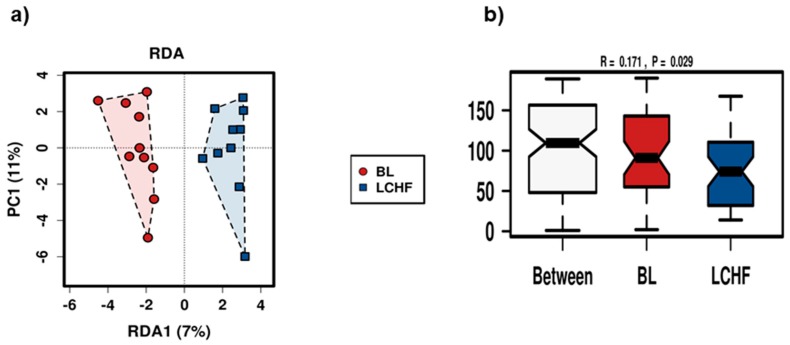
(**a**) Redundancy analysis (RDA) and (**b**) Anosim plots of the stool microbiota profiles for athletes either at baseline (BL, red) or after consuming the Low Carbohydrate High Fat (LCHF) diet (blue). In panel (**b**) the between plot is a measure of the magnitude of difference in profiles between the samples collected at BL and after consumption of the LCHF diet. Both analyses showed that the differences in the stool microbiota profiles between the two sampling periods are statistically significant (*p* = 0.005 and 0.029, for RDA and Anosim analyses, respectively).

The LCHF diet (*n* = 10 athletes) had a stronger impact on the stool microbiota profiles of the athletes than the HCHO and PCHO diets. RDA and Anosim both identified significant differences (*p* = 0.020 and *p* = 0.029 respectively) between the stool microbiota profiles of athletes at BL and following the consumption of the LCHF diet (Figure 4a,b).

**Figure 5:**
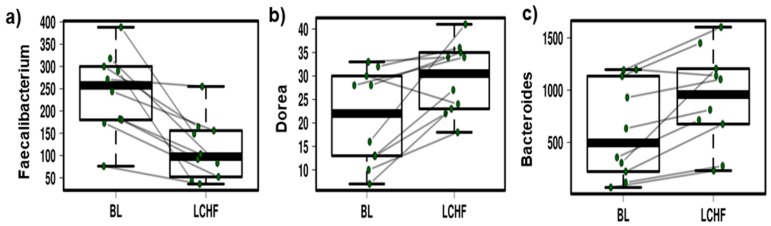
Mixed effect linear regression identified significant reductions in the relative abundances of *Faecalibacterium* ((**a**), *p* = 0.0003) and significant increases in the relative abundance of both *Dorea* ((**b**), *p* = 0.0068) and *Bacteroides* ((**c**), *p* = 0.0022) after consumption of the Low Carbohydrate High Fat (LCHF) diet. Here, sampling time point was set as a fixed effect and athlete as a random effect. Baseline (BL) and LCHF refer to the relative abundances of these taxa measured at baseline and after consumption of the LCHF diet, respectively. Those data collected from the same athlete are connected by the lines.

However, there was no significant change in the alpha diversity between BL and post LCHF consumption (Supplementary Figure S2). Furthermore, the LCHF diet resulted in a significant reduction in the relative abundances of *Faecalibacterium* spp. (*p* = 0.0003), an increase in *Dorea* spp. (*p* = 0.007) and several OTUs assigned to the genus *Bacteroides* (*p* = 0.002) (Figure 5a–c). These results were further verified using Wilcoxon rank *t*-test (*p* = 0.002, *p* = 0.01, *p* = 0.003 respectively) (Supplementary Figure S3).

**Figure 6:**
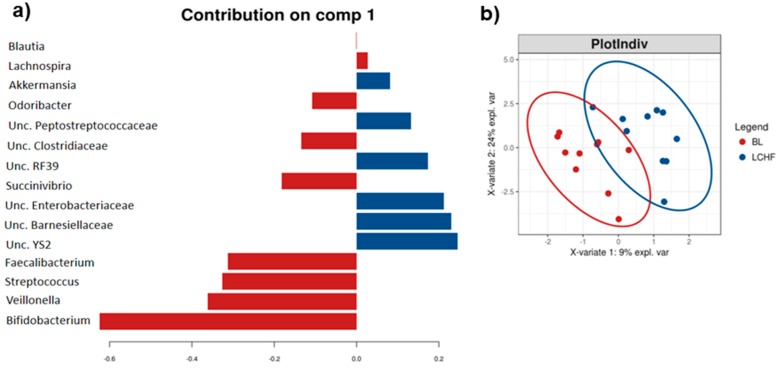
(**a**) Genera differentiating between the stool microbiota profiles of athletes at baseline (BL, red) and after their consumption of the Low Carbohydrate High Fat diet (LCHF, blue) identified by sPLS–DA; (**b**) The sPLS-DA ordination plot of these same data for each athlete, the ellipsoids represent 95% confidence intervals for each sampling period.

Analysis using LefSe also identified an increase of *Dorea* as well as *Enterobacteriaceae* in response to the LCHF diet and a reduction of *Faecalibacterium* and *Bifidobacterium* spp. (Supplementary Figure S4a,b). The sPLS-DA further found an increase in the relative abundance of OTUs assigned to *Unc. Peptostreptococcaceae*, *Unc. RF39*, *Unc. Enterobacteriaceae* and *Unc. Barnesiellaceae* and *Akkermansia;* while the relative abundances of *Bifidobacterium, Veillonella, Streptococcus, Faecalibacterium, Succinivibrio, Odoribacter* and *Lachnospira* spp. were reduced after consumption of the LCHF diet (Figure 6). In summation, these results suggest that the LCHF diet results in a stronger selective pressure on the gut microbiota of these athletes than the HCHO or PCHO diets, which were closer to their typical diets, leading to an increase in the relative abundance of bacterial taxa with recognized capabilities for lipid metabolism.

### 3.4. Bacteroides and Dorea spp. Abundances Are Negatively Correlated with Athlete Performance Measures Following Consumption of the LCHF Diet

**Figure 7:**
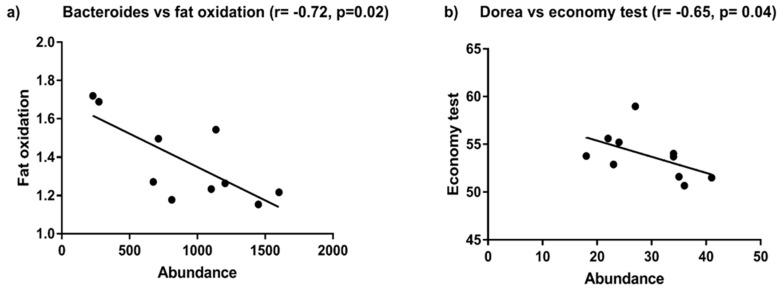
The relationship between (**a**) the abundance of *Bacteroides* spp. and fat oxidation and; (**b**) the abundance of *Dorea* spp. and economy test, for athletes after their consumption of the low carbohydrate high fat diet. The Spearman correlation coefficient and p values for each are also shown, and were statistically significant.

Based on the statistically significant differences in *Bacteroides, Faecalibacterium and Dorea* observed between the stool microbiota profiles of those athletes consuming the LCHF diet and their BL samples, these data were compared with various physiological and performance measures also collected at the beginning and the end of the diet-training period as part of the Supernova 1 study. Although no significant correlations were found between these taxa and any performance measures at BL, significant negative correlations (Spearman test) were apparent after consumption of the LCHF diet between *Bacteroides* abundance and fat oxidation (*r* = −0.72, *p* = 0.02); and *Dorea* spp. abundance and exercise economy (*r =* −0.65, *p* = 0.04, Figure 7).

### 3.5. Is There a LCHF × Enterotype Interaction?

Given the LCHF diet had the strongest impact on the faecal microbiome of athletes and there was a balanced distribution of athletes between the *Bacteroides* (*n* = 5) or *Prevotella* (*n* = 4) enterotype at BL, we also examined whether there was a diet × enterotype interaction evident in these athletes. Interestingly, while all the athletes did show a decrease in the relative abundance of specific *Prevotella* affiliated taxa and an increase in the relative abundance of specific *Bacteroides-*affiliated taxa (Supplementary Figures S5 and S6) these alterations were not sufficient to disrupt their “enterotype.”

**Figure 8:**
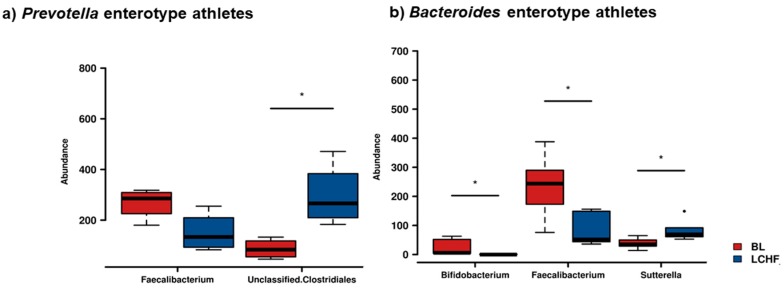
Rank testing of the changes in relative abundances of specific bacterial taxa between baseline (red) and following consumption of the Low Carbohydrate High Fat diet (LCHF, blue), when athletes are stratified according to their stool enterotype being either *“Prevotella-*dominant” (**a**) or “*Bacteroides-*dominant” (**b**). In athletes assigned to either enterotype, there was a reduction in the relative abundance of *Faecalibacterium* following consumption of LCHF diet. In those athletes with the *Prevotella-*dominant enterotype, a significant increase in *Unc. Clostridiales* was observed (*p* < 0.05); whereas a significant reduction in *Bifidobacterium* and an increase in *Sutterella* was observed in athletes with the *Bacteroides-*dominant enterotype following consumption of the LCHF diet (*p* < 0.05 in both instances). * represents significant differences (*p* < 0.05).

Rank tests revealed there was also a significant (*p* < 0.05) reduction in the relative abundance of *Faecalibacterium* following the LCHF diet intervention, independent of their BL enterotype. In contrast, *Bifidobacterium* was significantly reduced and *Sutterella* increased in those athletes with a *Bacteroides* enterotype, whereas *Unclassified* members of *Clostridiales* were significantly increased in the athletes with *Prevotella* enterotype (Figure 8). Taken together, these findings suggest there were also LCHF × enterotype interactions in this athlete cohort.

## 4. Discussion

Diet is now widely accepted as one of the major determinants of the composition and function of the gut microbiota, with concordant impacts on our nutrition and health. However, despite the robust evidence that diet is a critical factor in the metabolism and performance of endurance exercise/sport [15], very few studies have reported on the effect of specific dietary patterns during periods of intensified training on athlete physiology and the gut microbiota. Unfortunately, logistical constraints precluded the recruitment of a matching non-athlete (non-race walkers) cohort and the provision of comprehensive data on their habitual diet (i.e., prior to baseline). However, our study does provide the first in-depth investigations of the effect of dietary interventions for periods of intensified training on the gut microbiome of elite athletes and is empowered by the repeated measures for each athlete. This not only provides a direct comparison of their stool microbiome pre- and post-intervention but also the capacity to compare how any changes in the gut microbiome might be correlated with athlete performance measures. Thus, our study still provides valuable insights about the diet, microbiome and performance interactions. 

The baseline samples from this study provided some insight into the gut microbiota profiles of elite endurance athletes (race walkers) when consuming their habitual diet ahead of intensified training. Our results show that the stool microbiota of race walkers could be clearly separated into two principal “enterotypes”: one being *Prevotella*-predominant (7/29) and another *Bacteroides*-dominant (20/29). In general terms, these two “enterotypes” bear similarities with those originally proposed by Arumugam et al. [16] from their analyses of cohorts of both patient and healthy subjects drawn from the general communities of western European countries; the enterotypes were also reported in a recent study of amateur and professional level cyclists by Peterson et al. [7]. From their study of healthy non-athlete volunteers, Wu et al. [17] proposed that long term dietary patterns were the primary determinant of the persistence of either a *Bacteroides* or *Prevotella* enterotype, with diets favouring animal protein and fats supporting a *Bacteroides-*dominant enterotype; whereas a carbohydrate/fibre-rich diet favoured the establishment of the *Prevotella* enterotype. In that context, while Clarke et al. [5] did not report there being similar enterotypes to those noted above, the stool microbiota profiles of professional rugby players could be differentiated from the BMI-matched and normal BMI non-athletes by an increased relative abundance of taxa assigned to *Akkermansia, Succinivibrionaceae,* S24-7, RC9 and *Succinivibrio*. In our study, a single athlete could be separated from the two enterotypes noted above, because of the remarkable relative abundances of methane-producing archaea *(Methanobrevibacter)* and succinate-utilizing bacteria *(Succinivibrio),* as well as unclassified members of the phylum Tenericutes, order *RF39* and *Anaeroplasmatales.* It is still unclear as to extent which environmental, genetic or lifestyle factors might contribute to the presence of enterotypes in the human gut microbiome, as host physiological factors such as BMI, age and so forth, do not seem to be strong drivers of these profiles. However, Vandeputte et al. [18] recently revealed that the total bacterial load of the stool sample can be strongly associated with the enterotype predicted from the same stool sample. Furthermore, Korean et al. [19] have suggested that analytical factors such as the OTU picking method and the taxonomic level at which the data are studied, as well as the distance metric(s) and cluster scoring methods used, can also have some influence on data resolution and enterotype predictions. Here, our analytical methods clearly revealed the presence of enterotypes consistent with those reported for healthy mainstream subjects as well as other categories of elite athletes. Our analyses also suggest these enterotypes were resilient to change during the 3-week dietary intervention periods. Taken together, it therefore seems plausible that like in other healthy mainstream subjects, the stool microbiota enterotype of these elite athletes are resilient to short-term changes in their diet. Based on these results, future studies of elite athletes and/or where the number of subjects that can be recruited into the study might be constrained, a prospective assessment of the gut microbiome and enterotype representation be undertaken; to determine how subjects might be assigned to different interventions/treatments and to assess diet x enterotype interactions. 

Clarke et al. [5] reported significantly a greater relative abundance of *Akkermansia* in Irish rugby players as compared to the non-athlete controls and Peterson et al. [7] reported the presence of *Akkermansia* in 30/33 cyclists in their study. Here, OTUs assigned to the genus *Akkermansia* were detectable in some but not all the athletes and the different diets used here also appeared to have a limited effect on the relative abundance and/or prevalence of this genus. It is plausible that the difference in the relative abundance and/or prevalence of *Akkermansia* spp. between the specialist athlete groups could be linked with the differences in their dietary protein intake (16–17% daily caloric intake in race walkers, as compared to 22% in rugby players [5] and ~33% by most professional level cyclists [7]. However, future studies need to be conducted to establish this hypothesis.

The relative abundance of *Faecalibacterium* spp. was found to be decreased in athletes after their consumption of the LCHF diet. Interestingly, previous studies have not remarked on the relative abundance of this bacterium in athletes, despite it being one of the most abundant bacterial taxa present in the gut microbiota of healthy mainstream subjects. *Faecalibacterium prausnitzii* is also widely recognized for its production of a suite of metabolites and peptides with anti-inflammatory effects [20]. Studies with rodent models and human subjects of obesity and type 2 diabetes have shown reductions in the relative abundance of *F. prausnitzii* associated with these conditions. High-fat diets are likely to change the both the amounts and profile of bile acid secretions reaching the large intestine [21], which could also result in reductions in the relative abundance of *Faecalibacterium* spp., as it is known to be a bile sensitive bacterium [22,23]. Taken together, the significant reduction in the relative abundance of *Faecalibacterium* spp. in response to the consumption of the LCHF diet is both plausible and the potential effects of this change should be further investigated.

The comparative analyses also showed there was an increase in the relative abundance of *Dorea* spp. in response to consumption of the LCHF diet. This finding is consistent with those reported from rodent-based studies of obesity and lipid metabolism using high-fat diets [24,25,26]. Interestingly, positive associations have been reported between the relative abundance of *Dorea* spp. with serum total cholesterol and LDL concentrations in high fat-induced hyperlipidaemic rats [27]. Furthermore, while the relative abundance of *Dorea* spp. is consistently increased by high-fat diets, this change can be counteracted in rats by the supplementation of their diets with either mono- or tributyrin; and both compounds are also associated with reductions in liver and serum biomarkers of hyperlipidaemia [27,28]. Presently, much less is known about the possible roles of *Dorea* spp. in human obesity and lipid metabolism but our findings provide further evidence that such studies are warranted.

Despite the resilience of an athlete’s stool enterotype to short-term dietary change, there was a notable increase in the relative abundance of *Bacteroides* spp. in those athletes that consumed the LCHF diet. As noted above, high fat diets tend to increase bile acid secretion into the gut and David et al. [29] have reported an increase in the abundance of bile-tolerant bacteria in human subjects who consume a diet rich in animal-based proteins and fats and *Bacteroides* spp. are well recognized for their resistance to these host secretions. In that context, Wu et al. [17] have previously reported strong positive correlations between members of this genus and the intake of dietary fat- and protein-based nutrients as reported by questionnaire. Similarly, Shankar et al. [30] reported the gut environment of the group of US children studied was rich in metabolites arising from animal proteins and fats and *Bacteroides-*dominated; as compared to Egyptian children with greater concentrations of short chain fatty acids and fibre-degrading genes and a *Prevotella-*dominated microbiota. In the larger, parent study supporting the research presented here (Supernova 1), Burke et al. [9] found that although all dietary groups improved their aerobic capacity over the 3-week training block, the LCHF diet was associated with a negative effect on exercise economy and performance in the elite race walkers, when compared to those athletes consuming either the HCHO or PCHO diets. Interestingly but somewhat paradoxically, our analyses revealed that after the consumption of the LCHF diet, the relative abundance of *Bacteroides* spp. was significantly negatively correlated with fat oxidation; and that the relative abundance of *Dorea* spp. was significantly negatively correlated with the economy test, measured as described by Burke et al. [9]. Such findings suggest that an individual’s responsiveness to such a diet is complex and perhaps, can also be affected by the amount of dietary fat that actually reaches the distal gut, where it may have associative effects on the gut microbiota of the nature reported here.

In conclusion, this study has reported for the first time the stool microbiota profiles of elite endurance athletes bear gross similarities to those reported for healthy mainstream and other elite endurance athletes, in so far as the representation of *Bacteroides* and *Prevotella-*dominated enterotypes. While these enterotypes appear relatively stable in response to short-term changes in diet and despite the relatively small number of subjects available for study, a ketogenic low carbohydrate, high fat diet was still found to invoke significant alterations in the relative abundances of some key bacterial taxa. Although the findings of this pilot study cannot differentiate between causes versus consequence, the findings do justify the need for more detailed longitudinal studies that examine how diet × microbiome interactions may be better understood and managed to optimize athlete training and performance.

## Supplementary Materials

The following are available online at http://www.mdpi.com/2072-6643/11/2/261/s1. Figure S1: (a) Genera identified by LefSe analyses that differentiate between the stool microbiota profiles of athletes at baseline (BL, red) or after their consumption of the Periodised Carbohydrate diet (PCHO, blue); (b) Genera identified by LefSe analysis that differentiate between the stool microbiota profiles of athletes at baseline (BL, blue) and after their consumption of the High Carbohydrate diet (HCHO, red). Figure S2: (a) Shannon and (b) Simpson alpha diversity measures of the stool microbiota profiles for athletes at baseline (BL) and after their consumption of the Low Carbohydrate High Fat diet (LCHF). The diversity measures at the two sampling times were compared by mixed effect linear regression, including sampling time point as fixed effect and athlete as a random effect, and were not statistically different. Figure S3: Wilcoxon rank t-test identified significant reductions in the relative abundances of *Faecalibacterium* (*p* = 0.0003) and significant increases in the relative abundance of both *Bacteroides* (*p* = 0.0022) and *Dorea* (*p* = 0.0068) in athletes after consumption of the Low Carbohydrate High Fat (LCHF) diet as compared to their baseline (BL) profiles. Figure S4: Genera identified by LefSe analysis that differentiate between the stool microbiota profiles of athletes at baseline (BL, red) and after their consumption of the Low Carbohydrate High Fat diet (LCHF, blue) at either the OTU level (a), or at the genus level (b). Figure S5: Pie charts showing the genus level profiles of those athletes with the *Prevotella-*dominant enterotype at: (a) Baseline (BL) and (b) Post treatment (PT), i.e. after consumption of the Low Carbohydrate High Fat (LCHF) diet. The relative abundance of *Bacteroides* and *Unc. Clostridiales* were increased, whereas the relative abundance of *Faecalibacterium* was decreased in these athletes, PT. Figure S6: Pie charts showing the genus level profiles of those athletes with the *Bacteroides*-dominant enterotype at: (a) Baseline (BL) and (b) Post treatment (PT), i.e. after consumption of the Low Carbohydrate High Fat (LCHF) diet. The relative abundance of *Bacteroides* and *Sutterella* were increased, whereas the relative abundance of *Faecalibacterium* was decreased in these athletes, PT. Table S1: Anthropometric details of the subjects enrolled in this study.
