# Supplementary figures and images for: The Effects of Dietary Pattern during Intensified Training on Stool Microbiota of Elite Race Walkers
Source: Nutrients. 2019 Jan 24;11(2):261. doi: 10.3390/nu11020261 (PMC6413084; doi:10.3390/nu11020261)

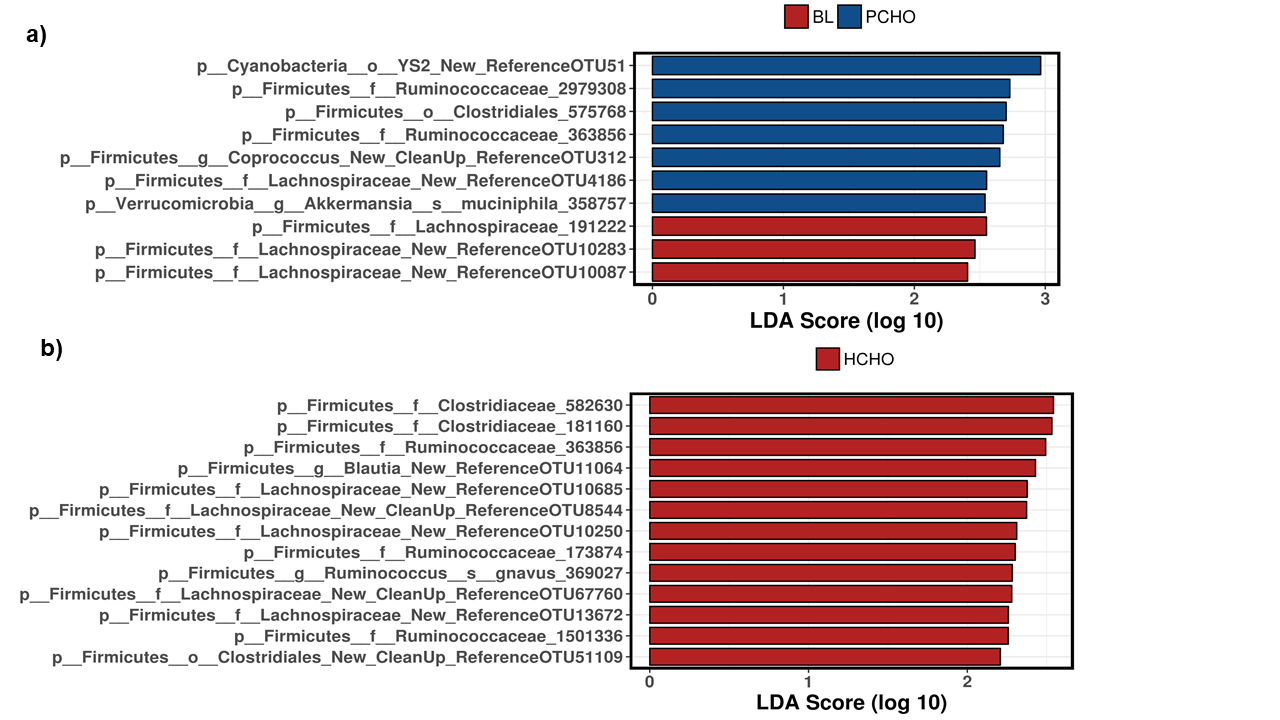

Supplement: Supplementary file 1 [file nutrients-11-00261-s001.zip › supplementary_files/S1.tif]

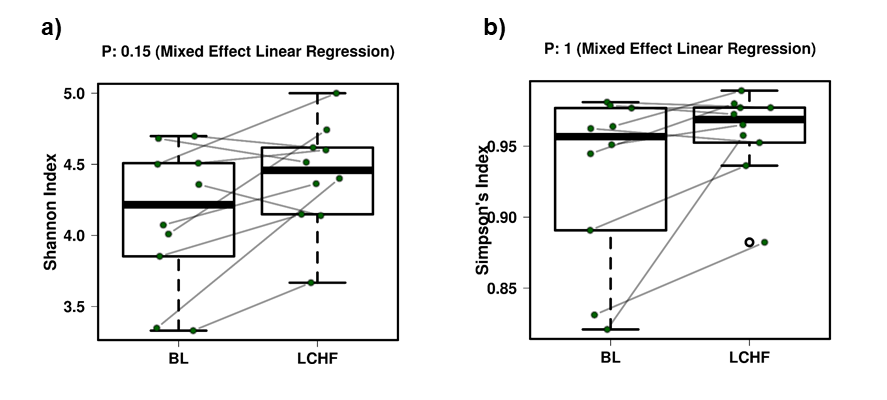

Supplement: Supplementary file 1 [file nutrients-11-00261-s001.zip › supplementary_files/S2.tif]

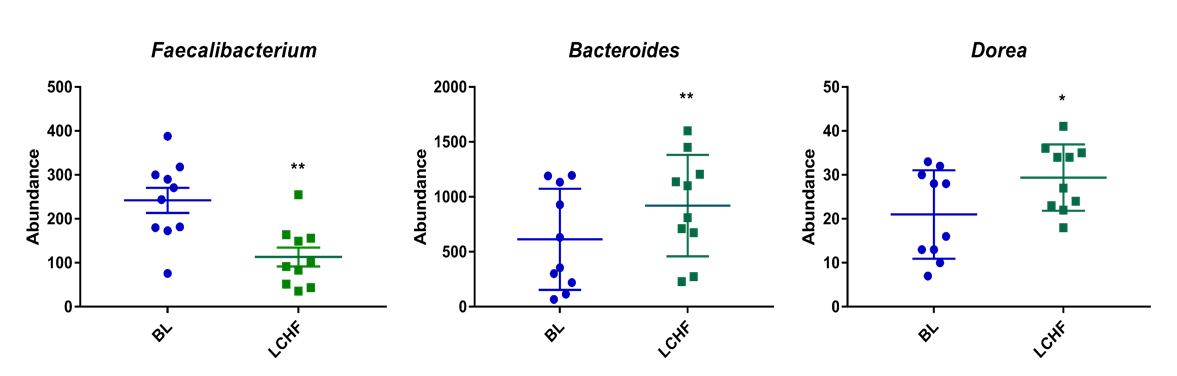

Supplement: Supplementary file 1 [file nutrients-11-00261-s001.zip › supplementary_files/S3.tif]

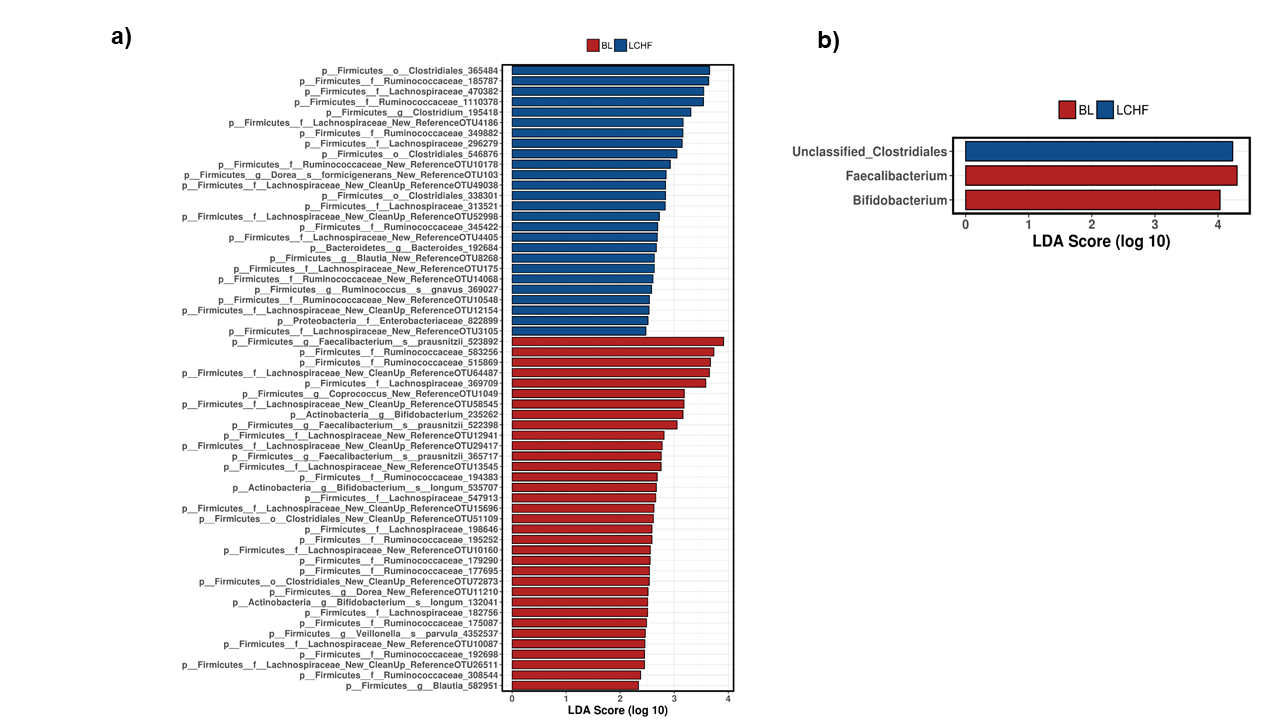

Supplement: Supplementary file 1 [file nutrients-11-00261-s001.zip › supplementary_files/S4.tif]

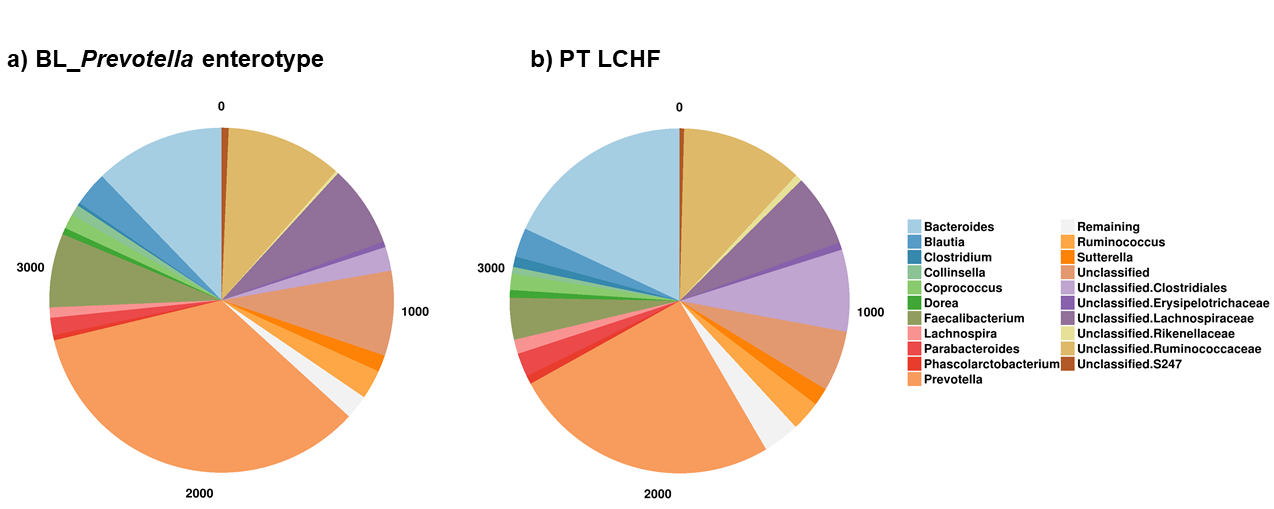

Supplement: Supplementary file 1 [file nutrients-11-00261-s001.zip › supplementary_files/S5.tif]

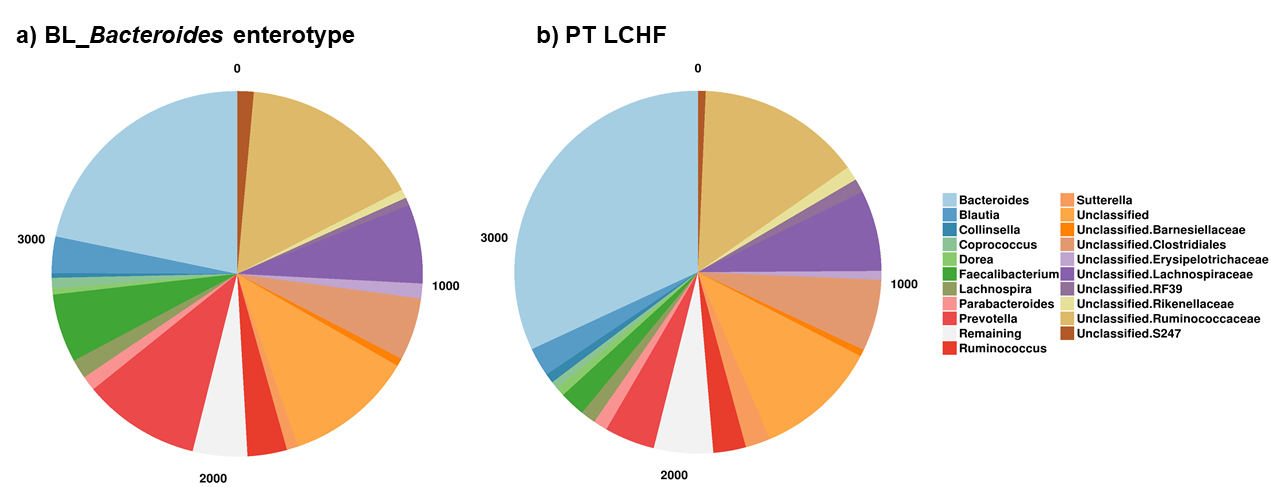

Supplement: Supplementary file 1 [file nutrients-11-00261-s001.zip › supplementary_files/S6.tif]
